# Classification and Treatment for Cervical Spine Fracture with Ankylosing Spondylitis: A Clinical Nomogram Prediction Study

**DOI:** 10.1155/2022/7769775

**Published:** 2022-03-04

**Authors:** Nana Shen, Xiaolin Wu, Zhu Guo, Shuai Yang, Chang Liu, Zhaoyang Guo, Shang-You Yang, Dongming Xing, Bohua Chen, Hongfei Xiang

**Affiliations:** ^1^Department of Rehabilitation, The Affiliated Hospital of Qingdao University, Qingdao 266000, China; ^2^Department of Orthopedics, The Affiliated Hospital of Qingdao University, Qingdao 266003, China; ^3^Department of Orthopedics, University of Kansas, School of Medicine-Wichita, Wichita, KS 67214, USA; ^4^Cancer Institute, The Qingdao University, Qingdao 266003, China

## Abstract

**Objective:**

Through the follow-up analysis of cervical spine fracture cases with ankylosing spondylitis (AS), a treatment-oriented fracture classification method is introduced to evaluate the clinical efficacy guided by this classification method.

**Method:**

A retrospective analysis was performed on 128 AS patients who underwent comprehensive treatment in the Spine Surgery Department of Qingdao University Hospital from January 2009 to May 2018. Statistics of patient demographic data, distribution of different fractures corresponding to surgical methods, 3-year follow-up outcomes, and summary of objective fracture classification methods were analyzed. A prospective 5-year follow-up study of 90 patients with AS cervical spine fractures from June 2015 to August 2020 was also included. Statistical differences on the distribution of factors such as case information, cervical spine sagittal sequence parameters, and fracture classification were assessed. Correlations between surgical information, American Spinal Injuries Association grade (ASIA), modified Japanese Orthopaedic Association scores (mJOA), and other factors were analyzed to establish a nomogram predictive model for curative effect outcomes. Overall, three major types and the four subtypes of AS cervical spine fractures were evaluated based on the clinical efficacy of the classification and the selection of surgical treatment methods.

**Result:**

The most common type of fracture was type II (30 cases, 33.33%), most of the subtypes were A (37 cases), followed by B (36 cases) and C (17 cases). Twenty-four of 28 patients with type I underwent anterior surgery, and 47 of 62 patients with type II and III underwent posterior surgery. The average follow-up time was 25.76 ± 11.80 months. The results of predicting clinical variables are different but include factors such as fracture type and subtype, type of operation, and age. The predictor variables include the above-mentioned similar variables, but survival is more affected by the fracture type of the patient.

**Conclusion:**

This predictive model based on follow-up information delineation points out the impact of ankylosing spondylitis cervical spine fracture classification on surgical selection and clinical efficacy.

## 1. Introduction

Ankylosing spondylitis (AS) is a progressive inflammatory rheumatic spondyloarthropathy affecting nearly 0.5% of the global population [1, 2]. The rigid segment becomes stiff; the lever arm deforms and is more likely to fracture following a small amount of force. It is estimated that the relative risk of AS vertebral fractures is thrice that of the global population. About 14% of AS patients experience a fracture in their lifetime [3, 4]. More than 80% of fractures in AS patients are associated with the lower cervical spine and the cervical-thoracic junction [1, 5]. Although various sophisticated surgical techniques have been developed to explore the treatment of cervical spine fractures in AS, the management of patients with AS may still be complicated by the presence of high risk of limited spinal motion, osteoporosis, potential clinical complications, or neurological injury [1, 3, 6, 7]. In addition, the incidence of cervical spine fractures in AS is low, and some patients even lose their lives early in the trauma due to direct spinal cord injury. The basis for clinical management of cervical fractures in AS is staging, but unfortunately, the classification is still ambiguous and tends to separate from actual clinical observations. So far, there is a lack of cumulative clinical predictive analysis and classification-related surgical methods and outcome selection criteria [8]. This adds uncertainty to the effective intervention of AS cervical spine fractures and surgical expectations. Also, there are often unexpected results and improper surgical applications caused by the “the injury of both people and money” phenomenon.

In this study, we adopted the AS cervical spine fracture classification method based on the combination of surgical experience and clinical surgical observation. We considered both a neurological condition and a radiological assessment [2]. With the accumulated cases, we attempted to analyze and predict the treatment situation under the new classification by reviewing the previous treatment expectations. This provided a new basis for the clinical treatment of AS.

## 2. Materials and Methods

### 2.1. Retrospective Demographics

We retrieved 128 inpatient and outpatient electronic medical records from January 2009 to May 2018 in Qingdao University Affiliated Hospital for cervical spine fractures with imaging diagnosis of AS. All patients were hospitalized to have received surgical cervical spine interventions or cervical rehabilitation treatment. Data collected included basic statistics of patients, symptoms, and intervention options. This leads to a summary of the types of surgery and fracture types. This study has been approved by the Medical (Ethics) Committee of the Affiliated Hospital of Qingdao University (QDFY WZ 2015-15-07), and all participating patients are in compliance with ethical standards.

### 2.2. Prospective Demographics

We conducted a prospective 5-year follow-up study on 90 patients with AS cervical spine fractures from June 2015 to August 2020. The hospital ethics committee approved each intervention and research project. Before the operation, the patients were treated according to the new classification summarized through retrospective analysis. Patients signed an informed consent statement either by themselves or by family members. Data collected included patient case information, imaging cervical spine sagittal sequence parameters, and fracture classification. Results included surgical information, ASIA score, mJOA score, and so on.

### 2.3. Inclusion and Exclusion Criteria

For prospective studies, inclusion and exclusion criteria are established. The inclusion criteria were patients with clear clinical evidence of AS combined with cervical fracture, and all study data were recorded in the hospital electronic information system. The clinical diagnostic criteria for ankylosing spondylitis are: (1) restricted chest expansion with a maximum difference between expiration and inspiration of less than 2.5 cm; (2) sacroiliac arthritis seen on X-ray, bilateral grade II, unilateral grade III, or higher; (3) restricted range of motion in three directions, including forward bending, backward bending, and lateral bending of the lumbar spine; and (4) painful stiffness in the lower back for more than three months that does not improve with rest. The diagnosis of ankylosing spondylitis can be confirmed if one of the fourth plus 1 to 3 items is present. Patients with an unconfirmed AS diagnosis, upper cervical fracture, and significant spinal deformity were excluded, as were patients with injuries sustained earlier during the disease progression when the spine was still flexible [9]. All the patients were evaluated using X-rays, CT, and MRI before surgery to describe the circumstance of the injury site and the details of the spinal cord.

### 2.4. Classification and Treatment of Fracture

CT scan was used as an important examination for fracture staging, using 128-row medical spiral CT equipment (GE, Milwaukee, Wisconsin, USA), setting scan parameters: tube current, 200 mA; layer thickness and reconstruction interval, 5 mm and 5 mm, respectively; display field of view (DFOV), 20 cm; interval, 0.531:1; pixel interval, 0.430 mm; and spiral transient switching between 140 kVp and 80 kVp. Image reconstruction and analysis were performed using an advanced workstation (AW 4.7; GE Healthcare, USA), and all organ unit scans were performed according to routine procedures for scanning major pathological units, with informed consent from the family and the patient himself and ethical approval on file by the hospital.

**Figure 1:**
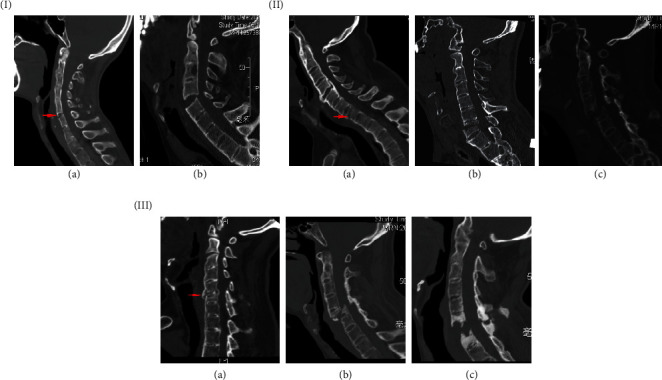
Fracture classification.

Prior to further treatment, the fracture was classified into three types based on the fracture line and severity: type I, disc injury; type II, vertebral body injury; and type III, vertebral body and disc injury. Four subtypes were also defined as follows: (A) fracture without dislocation, (B) fractures with dislocation without obvious bone defects, (C) fractures with obvious dislocation or severe bone gap, and (D) fractures with epidural hematoma or CSF leakage (Figure 1). Pre- and intraoperative skull tractions were used to immobilize the spine especially when the fracture was unstable.

All these patients underwent intraoperative neurophysiologic monitoring (IONM) including sensory and motor-evoked potentials during surgery [10]. Patients with incomplete neurological deficits were treated urgently (within 24 hours), whereas those with complete and central cord syndrome were surgically treated at a later time [2]. Surgical procedures were determined according to the different individual factors, such as fracture location, numbers of involved segments, fracture types, neurological deficits, among others.

Anterior decompression and fusion (AF) were conducted on at least one segment above and below the fracture site if anatomical access was permitted. A tricortical iliac graft was placed into the disc or fracture defects, and a plate internal fixation was then performed [5]. The posterior approach (PF) was conducted with long segments fixation in cases of instability, using the Mayfield head holder for traction and locking [8]. A combined laminectomy was performed on cases involving neurological deficit or epidural hematoma.

Halo-vest, sterno-occipital mandibular immobilization device (SOMI), or cervical collar was given to obtain cervical immobilization for 1–1.5 months after surgery.

All surgeries are performed by a unified team of six surgeons of equal level and appropriate rank and clinical background, and more importantly, the surgeons are fully executed intraoperatively with a consensus team preoperative discussion as the established plan for the surgery.

### 2.5. Evaluation Index

The clinical follow-up examinations were performed up to 5 years postoperatively. CT scans and X-rays evaluations were taken at each follow-up. The clinical outcomes were assessed using the ASIA grade and mJOA score. The ASIA grading scale is a neurological assessment of spinal cord injury developed by the American Spinal Cord Injury Association (ASIA), which classifies spinal cord injuries into grades A–E, with the degree of injury decreasing as the grade increases, focusing on the evaluation of neurological structure and function, while the modified Japanese Orthopaedic Association cervical spinal cord scoring system (mJOA) focuses more on the clinical evaluation of neurological function, with a total score of 18 points, involving the motor function of the upper extremity (5 points), lower extremity (7 points), sensation (3 points), and urination (3 points), with lower scores indicating more severe disability and impairment of spinal cord function. Secondly, injury site, fracture patterns, surgical procedures, fixation levels, operation time and blood loss, fusion rate, and complications were also documented. Complications were categorized as general (such as infection, respiratory failure, or death) and surgical (such as early implant failure or screw loosening).

Imaging measurements are measured by standard X-rays in the corresponding position, and the measurement parameters and methods are: (1) C_2–7_ COBB (°): C_2–7_ COBB's angle is the angle between the lower endplate of C_2_ and the upper endplate of C_7_ (anterior convexity is negative), (2) cSVA (mm): cervical sagittal vertical axis is the C_2_ vertebral body midpoint vertical axis to the distance of C_7_ vertebral body posterior superior angle, and (3) *T*_1_ slope (°): *T*_1_ tilt angle is the angle between the tangent line of the upper endplate of *T*_1_ vertebral body and the horizontal plane.

### 2.6. Statistical Analysis

Continuous data were presented as the mean ± standard deviation or median, the interquartile range, whereas the categorical data were presented as counts (percentages). According to the specific results, the sample size was calculated dependent on the regional incidence rate. Due to the five-year follow-up, all subjects had quality of life follow-up results. Multivariate logistic regression was used to establish a binary outcome prediction model, while multiple variables were used to establish a numerical outcome prediction model linear regression. The candidate variable of each model was a screening step where the *P* value was less than 0.3 in the univariate analysis. For the relaxation of the linear assumptions of numerical predictors, we used a restricted cubic spline function model [11]. Calibration was conducted by plotting the predicted patient proportion for each outcome and developing the actual proportion sample (obvious) and guide sample (bias correction) on each outcome for the original outcome. Appearance closely following the 45° equivalence line (ideal line) indicated high model calibration. For the numerical results, each final model reached the maximum deviation correction consistency correlation coefficient (pressure reduction CCC R software package). It represents the model fit through the adjusted coefficient of determination (*R*^2^).

## 3. Results

### 3.1. Descriptive Data

Of the 128 patients, 90 (81 males and 9 females) were enrolled and subjected to follow-up consecutively. Their mean age was 52.1 ± 10.4 years (range, 29–77). Three patients (3.33%) had 1 vertebral body or disc involved, whereas 87 patients (96.67%) involved 2 or more bodies. Notably, C6, C7 body, and C6-C7 disc were the most frequently involved (84 patients, 93.33%).

### 3.2. Surgical Approach Related to Classification

**Figure 2:**
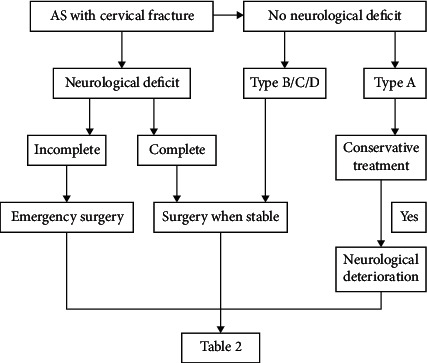
Treatment basis diagram.

Here, 82 patients underwent surgery according to our classification except for one patient who was subjected to revision surgery (AF + PF) because of early AF failure, and another three patients who underwent posterior surgery combined anterior surgery (PF + AF) owing to a sizeable anterior gap after posterior fixation (Figure 2).

An anterior-only approach was performed on 31 patients including the 1 patient mentioned above who underwent anterior surgery firstly, followed by AF + PF for revision. Besides, 48 patients underwent a posterior-only approach. The fixation segments related to classification are summarized in Table 1.

In type I (a total of 28 patients), 24 patients underwent anterior surgery, whereby 1 patient needed revision surgery after AF, while posterior surgery was performed on one patient. In type II (a total of 30 patients), 27 patients underwent posterior surgery, whereas anterior surgery was performed on 1 patient (type IIA). In type III (total 32 patients), 20 patients underwent posterior surgery with the fixation on more than 3 segments, whereas anterior surgery was performed on 6 patients; in addition, three patients received PF + AF. In addition, we have followed the principle of individualized surgical protocols, giving priority to: (1) maximum release of the spinal cord injury, (2) least traumatic and most mechanically stable fixation, and (3) best survival expectations and postoperative needs.

Using the anterior-only approach, the operation time was 130 ± 41.7 minutes (range: 80 to 305 min) on average and 185.9 ± 46.5 minutes (range: 110 to 280 min) in the posterior-only approach with significant difference (*P* < 0.001). Intraoperative blood loss during the anterior-only approach was 177.6 ± 138.0 cc on average (range: 50 to 800 ml), and 494.4 ± 313.6 cc (range: 100 to 1500 ml) in the posterior-only approach with significant difference (*P* < 0.001). There was a statistical difference in operation time and blood loss among type I, II, and III groups (*P*=0.020 and 0.027, respectively; Figure 2). The average operation time was 140.6 ± 49.7 minutes in type I, 178.3 ± 37.5 in type II, and 176.3 ± 62.4 in type III. In type I, the average blood loss was 231.4 ± 247.5 cc, whereas it was 485.8 ± 237.3 in type II and 447.5 ± 336.2 in type III. However, there was no statistical difference in average operation time and blood loss among subtype A, B, and C groups (*P*=0.534 and 0.444, respectively).

### 3.3. Distribution of Fracture Types in Retrospective Analysis

**Figure 3:**
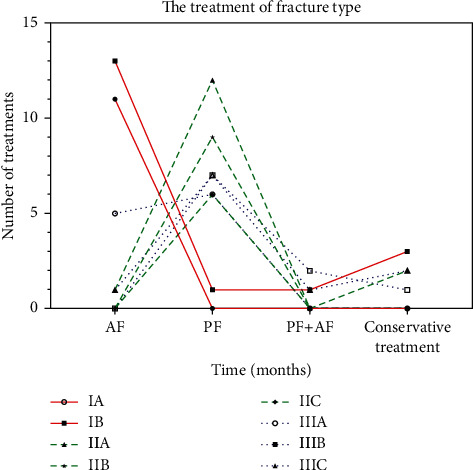
Statistics of fracture types and treatment methods.

According to our classification, different type and subtype of these fractures were recorded, including type I (28 patients, 31.11%), type II (30 patients, 33.33%), and type III (32 patients, 35.56%). The most common fracture pattern was type IIA (15 patients, 50%), and the majority of subtype was A (37 patients), followed by B (36 patients) and C (17 patients; Figure 3).

### 3.4. Basic Information in Prospective Research

In the prospective analysis, a total of 90 patients were selected, including 81 males and 9 females. The specific information of these 90 patients is given in Table 1.

### 3.5. Classification and Predictors of Treatment Outcomes

**Figure 4:**
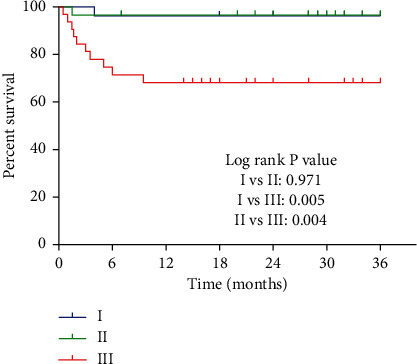
Prediction of clinical outcomes of main classification (survival curve).

Prediction of clinical outcome was mainly classified as follows (Figure 4): the patients who were followed up for 36 months were classified according to the main classification, and the survival curve was generated according to the survival situation of the patients. It is evident that the survival rates of type I and II patients in the main classification are basically the same; there is a slight decline in the first 6 months, whereas the survival rate remains unchanged during the remaining time (Figure 4). However, the survival rate of type III was significantly different from the first 2 types. In the first 12 months, the survival rate of patients of this type dropped sharply, especially in the first 6 months; the decline was more obvious. After 12 months, the patient's survival rate remained constant at about 60%, which was much lower than that of type I and II patients. Comparing the survival rates of the 3 types of fractures in pairs, there was no significant difference between types I and II (*P* > 0.05), while type III was highly significantly different whether it is type I or II (*P* < 0.05).

**Figure 5:**
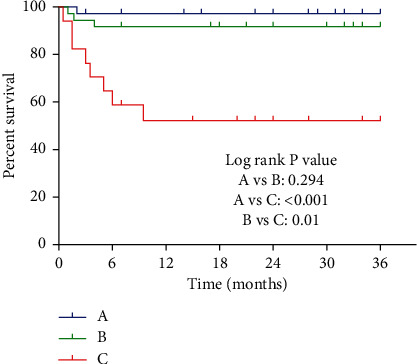
Prediction of subtype clinical outcomes (survival curve).

Prediction of subtype clinical outcome (Figure 5): the 36-month follow-up patients were classified according to subtypes, and survival curves were made according to the survival conditions of patients. By drawing a survival curve based on subtypes, we reported a certain degree of difference in the survival rates of the 3 types of patients. The survival rate of type A patients dropped slightly in the first 3 months; the survival rate of type B patients dropped to a certain extent within 6 months; and the survival rate of type C patients dropped significantly within 12 months. The survival rate of patients with types A and B was relatively small (*P* > 0.05) but could be maintained above 90%, and the main decline time was in the first 6 months. However, the survival rate of type C decreased significantly in the first 12 months, especially in the first 6 months where the survival rate of patients dropped sharply. Besides, the survival rate of type C patients was maintained at about 50% after 12 months, which was much lower than types A and B. Notably, the survival rate of type C patients was significantly different from that of type A or B (*P* < 0.05).

**Figure 6:**
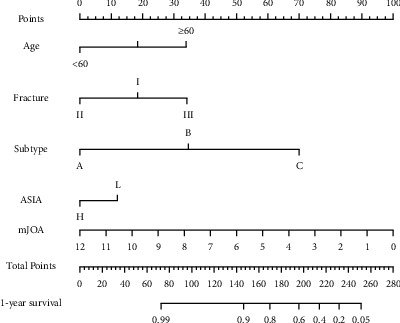
Predictive evaluation nomogram for fracture classification and clinical evaluation.

**Figure 7:**
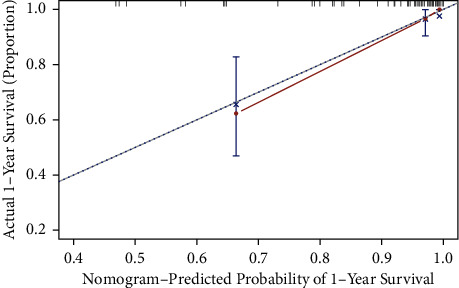
Calibration curve for one-year survival prediction.

Based on the above results, we constructed a predictive evaluation nomogram for fracture classification and clinical evaluation (Figure 6), which was validated using a calibration curve (Figure 7). The deviation-corrected c-index of the one-year survival rate was 0.63. For the QOL index, *R*^2^ = 0.4 after deviation correction adjustment.

### 3.6. Predictors of ASIA

After analyzing data of the collected cases, 13 factors such as gender, age, and BMI were compared. Compiled results are presented in Table 2. The data mainly elucidated the type of fracture risk and 95% confidence interval. Thereafter, the obtained data were calibrated using a uniform standard to obtain the most valuable results with the smallest variables. To evaluate the impact of the ASIA score on the occurrence and development of fractures, the surgical method was inferred.

Notably, factors including gender, smoking or not, COBB angle, and *T*1 tilt rate were not statistically significant in the risk of fracture (*P* > 0.05; Table 2). The difference between BMI and ASIA scores may be attributed to the different risks of fracture (*P* < 0.05). A larger BMI index indicated a worse ASIA score, which increased the probability of fracture. Moreover, the ASIA score was more closely related to the occurrence of fractures and had a higher correlation. Of note, the 95% confidence interval of the ASIA score was 3.79–433.73, which could correlate the fracture classification with the ASIA score.

### 3.7. Predictors of mJOA

Similar to the ASIA score, the factors related to the mJOA score are presented in Table 3. Among them, gender, COBB angle, *T*1 tilt rate, and fracture type and risk were not statistically significant (*P* > 0.05). Moreover, mJOA had a high correlation with the type and probability of fracture (*P* < 0.05). Similarly, in comparison to the data related to the ASIA score, BMI was not statistically significant at this time (*P* > 0.05). Although slight differences in specific values were reported (Tables 2 and 3), the overall trend was the same. This demonstrated that the type of fracture is not only related to the ASIA score but also to mJOA. The 95% confidence interval of mJOA was 0.5–0.84 (see Table 3). Thus, we speculated that this would be more accurate in predicting the type of fracture and the risk of occurrence. Compared with other factors, the significance of mJOA in the data may be more significant. Through a comprehensive comparison, in the fracture classification, the ASIA score may also have a certain correlation with mJOA.

### 3.8. Cox Regression Analysis of Fracture Predictors

Through COX regression analysis, we revealed the factors that may be attributed to different fracture types (Table 4). Among them, age, ASIA score, and mJOA score significantly had a huge impact on the predictive factors of fracture (*P* < 0.05). There is no doubt that age had a definite influence on the occurrence of fractures. With the increase of age, especially the elderly above 60 years, the risk of fracture inevitably increased, and the type of fracture was more severe.

At the same time, the role of the ASIA score and mJOA score in the types of fractures could not be ignored. As the degree of fracture worsened, severe spinal cord injury inevitably led to more obvious sensorimotor disorders. We validated this phenomenon using COX regression analysis. Results demonstrated that ASIA score and mJOA score were important predictors; thus, their influence cannot be ignored, and they were highly correlated. Among them, the 95% confidence interval of the ASIA score was 1.48–88.85, whereas that for the mJOA was 0.47–0.83, indicating their highly significant correlation with the type of fracture.

## 4. Discussion

### 4.1. Cervical Fracture Characteristics and Classification Related to AS

AS, which is a chronic disease, typically starts before the age of 30 with a slow but steady progression [11, 12]. In the present study, patients suffering from AS for an average of 25–28 years at the time of injury [5, 13] were enrolled for analysis. Notably, the age distribution indicated that cervical fracture in AS is, in most cases, associated with patients between the age of 40 and 60 years (58 patients, 71.60%). Previous reports demonstrated that 75% to 81% of cervical fractures with AS involved lower cervical spine (C5–C7) [3, 14], a finding that was nearly consistent with our results.

Due to the long lever arms and biomechanics of the ankylosed spine, the classical three columns were not applicable for managing cervical fracture in AS patients [8, 15]. Although the new AO spine fracture classification system introduced the modifier M2 to mark the severity of the fracture with AS, it is only applicable for thoracolumbar fracture [16]. Recently, three classifications have been developed concerning AS-related cervical fractures [4, 9, 17].

Metz-Stavenhagen et al. described two subtypes for cervical fracture in AS: type I, the complete disruption of anterior and posterior bony and ligamentous structures, and type II, the sintering fracture, often after a minor injury, unnoticed by the patient [8, 9]. Elsewhere, de Peretti et al. described a classification of four fracture types according to radiographic dislocation: type I with anterior opening, type II with horizontal dislocation, type III non-displaced, and type IV being similar to spinal fracture and unrelated to AS6. In addition, the classification introduced by Caron et al. involved the radiographic course of the fracture line (type I, disc injury; type II, body injury; type III, anterior body or posterior disc injury; and type IV, anterior disc or posterior body injury). Collectively, these classifications remained academic, and no impact of fracture type on patient treatment or outcome has been described until now [8]. Thus, we assessed the radiographic fracture severity and presented a new classification for further treatment and prediction of outcomes. The classification was as follows: type I, disc injury; type II, body injury; and type III, body and disc injury, and three subtypes were added (A, fracture without dislocation; B, obvious dislocation without bone defects; and C, obvious dislocation and bone defect in the vertebral body). Types I, II, and III presented the transverse diaphyseal long bone fracture with different fracture lines, whereas subtypes A, B, and C revealed cervical fracture severity complicated with dislocation or bone defect in the vertebral body. We also revealed that type III and subtypes B and C may be the most unstable patterns, which should be taken into thoughtful consideration before the surgical approach; however, fixed segments were chosen.

In addition, this study combined the characteristics of the three original typologies to combine clinical prognosis and spinal cord functional recovery, presenting not only the anatomical characteristics of AS cervical fractures but also taking into account the risk factors of spinal cord injury, making the typology closely related to the choice of treatment, which is the advantage of this study's typology.

### 4.2. Choice of Treatment Related to Different Fracture Classifications

Treatment for this kind of fracture was controversial. It has been described that the fracture without dislocation or neurological deficit may be the gold standard for conservative treatment. This typically involved bed rest, axial traction, and immobilization with halo-vest [5]. Once an unstable cervical fracture was confirmed, patients could be managed with axial traction or through immobilization [18]. Of note, these conservative treatments were associated with significant problems: risk of skin ulcerations, local septic, and respiratory problems, worsening of the regional kyphosis with loss of reduction, risk of non-union because of the shearing forces on the fracture site [19], and risk of neurological aggravation [20]. Overall, we suggest that conservative treatment only may not be suitable for this kind of fracture, particularly, in patients with severe neurological symptoms and unstable patterns such as type III and subtypes B and C. Furthermore, we strongly recommend surgery for a cervical fracture in AS, which is presently widely used [11, 21–26]. The procedures had been described including anterior approach, posterior approach, and combined approach, and the surgical procedures in relation to classification were analyzed as described below.

### 4.3. Anterior Approach

Although the anterior approach may pose less trauma, blood loss, and operation time and minimize risks of displacement during positioning and postoperative infections [15], the anterior-only approach was not recommended for transverse, rotationally unstable fractures in AS [27]. Beyond that, many patients with AS were kyphotic, and the anterior access was anatomically impossible especially when the fracture was located at the cervicothoracic junction [8]. Therefore, the inferiority of the anterior-only surgery was reflected by the finding from several previous studies, in which implant failure occurred in the anterior-only treated patients [28, 29]. For instance, Kouyoumdjian et al. [5] suggested that anterior plate fixation may provide sufficient stability if the hardware is long enough to avoid significant moment arms.

**Figure 8:**
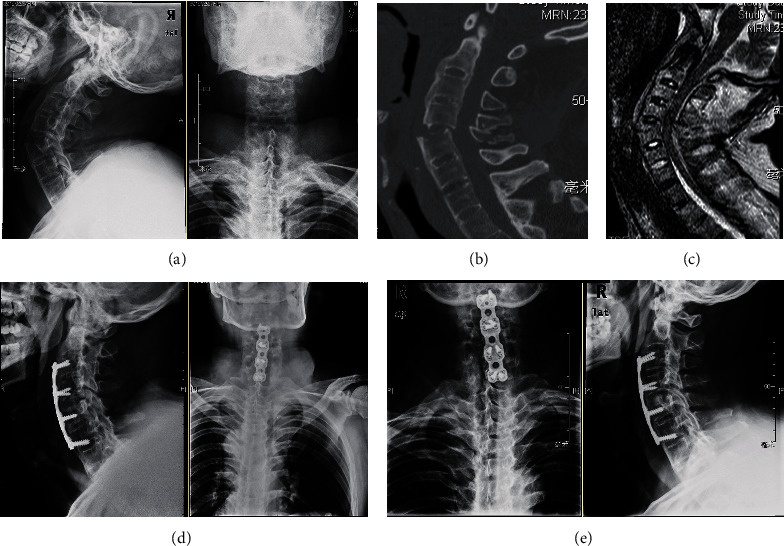
Type I fracture. An anterior approach is adopted. The length of the plate should be extended by more than two segments.

In our series, 36 patients underwent an anterior-only approach, and most of them were type I (30 patients, 83.3%) with transdiscal fracture, located between the former endplates. We considered that this type of fracture may preserve the bone stock of the anterior column with fair contact between the fragments without adding the anterior graft. Although we tried a shorter fixation in patients with the mild transdiscal fracture (type IA) who were treated with SOMI postoperatively for additional immobilization and showed satisfactory outcomes (Table 1), we still recommended a longer segment fixation in type I in case of implant failure (Figure 8).

### 4.4. Posterior Approach

Multilevel posterior-only approach for lower cervical fracture seemed biomechanically reasonable, even if the posterior approach may have considerable bleeding and more risk of infection. Posterior-only fixation was strong and stable with few implant failures, and the fixed region was sufficient with two segments above and two below the fracture segment [30, 31].

**Figure 9:**
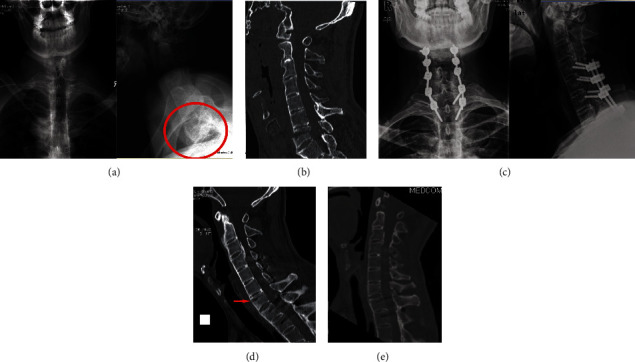
Treatment of type IIA; most fracture patients can be treated with posterior surgery, which is safer.

In our series, 42 patients underwent posterior-only approach, most of them were treated with long-segment fixation (39 patients, 92.9%), and only 3 patients in type IIA were treated with fixation equal to or less than 3 segments (Table 1). We have found that posterior fixation alone for lower cervical fracture was sufficient to obtain fusion if the fixation was long enough (Figure 9). Postoperatively, an additional cervical collar was mandatory with long-segment fixation, whereas SOMI for immobilization was initially preferred in patients with shorter segment fixation [18].

### 4.5. Combined Approach

A combined approach may be necessary only when the structural integrity of the vertebral body has been significantly compromised. Circumferential fusion should be a suitable method for these reasons: three-column instability, poor bone quality, and severe kyphosis [28].

In our experience, posterior decompression or fixation was performed combined with the anterior surgery when posterior compression and instability or epidural hematoma were noted on MRI with persistent neurological deficits after anterior surgery, or revision surgery was needed [5]. Similarly, anterior decompression and fixation were conducted after posterior surgery when anterior compression, significant instability, or severe anterior bone defect was revealed; notably, neurological deficits did not completely regress after posterior surgery. In our series, we conducted revision surgery (AF + PF) in one patient with early implant failure. Posterior surgery combined anterior surgery (PF + AF) was performed in three patients who underwent an anterior autologous iliac bone interbody fusion because of a sizeable anterior gap after posterior surgery.

In a nutshell, we do not recommend a one-stage combined approach as the first choice owing to increased blood loss, operation time, and complication. Thus, we preferred a unique approach most of the time. In type I, we preferred an anterior-only approach (＞3 segments) if anatomical access permitted. In types II and III, we recommended the posterior-only approach (＞3 segments) as the first choice. However, if long posterior instrumentation was performed, the anterior access became obsolete, since stabilized fractures related to AS had a tendency to heal, even if slight anterior defects were present. The fixation allowed early rehabilitation with molded collar or SOMI for stronger immobilization postoperatively. Thus, we preferred the anterior-only approach in type I most of the time, whereas the posterior-only approach in types II and III, and fixations were long but not systematically circumferential.

### 4.6. Outcomes and Complications

In our series, the criteria for determining bone fusion on CT are: (1) the presence of bridging bone trabeculae around the fracture line on the thin scan, (2) the presence of bridging bone trabeculae through the fusion device, (3) the presence of the above fracture healing signs in at least two vertical null straight scan planes, and (4) the presence of both of the above. All living patients achieved bone fusion confirmed by CT scan at last follow-up and improved or maintained their neurological status except for three patients who suffered deterioration in neurological status after surgery. The fracture subtype related to fracture severity may be predictive of neurological status and outcomes. Also, patients with subtype C may have more severe neurological symptoms and poor recovery, followed by patients with subtypes B and A. This indicated that subtypes may be related to neurological deficits and outcomes.

Moreover, AS Patients with cervical fractures were extremely prone to complications after surgical intervention. In another study, Einsiedel et al. [28] revealed that early implant failures occurred exclusively after single-session anterior stabilization (50%). In our series, two patients developed early implant failure or screw loosening after anterior stabilization alone. Implant failure may be attributed to difficult anatomy and osteoporosis of the spine and the surgeon's misunderstanding of biomechanics. For such predictable implant failures, we have taken compensatory measures in the form of adjunctive external fixation in all postoperative cases and opted for compensatory measures in the form of reoperation for endograft failures that may endanger neurological function.

As with other published reports [1, 5], the overall mortality rate in this injury was higher (33%), and related to the initial medullary involvement, the death in our series was in one patient (type IIIC, ASIA B before the surgery), who had significant medulla injuries visible on MRI. The patient was aged above 70 years with poor condition and died of respiratory failure and infection. These fetal complications may necessitate making difficult decisions regarding postoperative immobilization to avoid chest compressions and significantly interfere with the surgical strategy [18].

In the present study, the obvious epidural hematoma was identified on neuroimaging or during operation in nine patients (Figure 1), with a higher risk than in the non-AS population. All these patients presented with severe neurologic deficits (ASIA A or B). Although they experienced an improvement in their clinical status after surgery, there were still severe neurological deficits at the last follow-up. Thus, the hematoma could be a key factor in AS patients with cervical fracture as being predictive of severe neurological deficits and poor recovery.

### 4.7. Limitations

The limitations of this study are as follows: (1) the partial time overlap between the retrospective and prospective studies resulted in some patients appearing in different studies, and there may be a small bias in the summary of experience in the time frame; (2) the postoperative medical treatment of ankylosing spondylitis fractures may have an impact on the prognosis and partially influence the experimental results; and (3) the uncontrollable out-of-hospital rehabilitation and the uneven postoperative rehabilitation exercise methods had some influence on the clinical efficacy assessment of this study, which needs to be further extended and controlled by improving the experimental follow-up methods.

## 5. Conclusions

The present study demonstrated that patients with AS are highly susceptible to cervical fracture and extensive neurological injury caused by even mild traumatic force. X-ray, CT, and MRI imaging were strongly recommended regardless of whether minor initial clinical findings are present. Since conservative treatment alone is inadequate for this kind of fracture, we assessed the severity of the fracture based on radiological findings and presented a new classification to assist surgeons in their efforts to provide optimal surgical treatment.

Notably, the anterior-only approach is preferable in type I as it presents satisfactory results, whereas the posterior-only approach in types II and III, and fixation is long but not systematically circumferential. Also, the fracture subtypes especially B and C often indicated a more severe neurological status. It was revealed that S patients with a cervical fracture are extremely prone to complications after surgical intervention, which is related to the severity of the initial neurological presentation, and the epidural hematoma may be a key factor in AS patients with cervical fracture as being predictive of severe neurological deficits and poor recovery.

To better show our improved fracture classification, a nomogram was introduced in data analysis. The nomogram as an important data analysis tool may be of great significance to clinical research. This analysis method has been applied in many fields such as oncology and cervical diseases. It enables clinicians and patients to choose more reasonable treatment plans for specific diseases in a systematic manner based on data [32, 33].

Unfortunately, there was still no high-level evidence to guide the treatment, and the current data was based on our sentinel experience and small cases. Patients with obvious kyphosis or deformity were not in our series; thus, further studies are warranted to confirm these early findings.

## Figures and Tables

**Table 1:** Basic information.

Variable	*N* (%) or median (IQR)	Total number
Sex		90
** **Female	9 (10%)	
** **Male	81 (90%)	

Age		90
** **≥60	20 (22.2%)	
** **<60	70 (77.8%)	

BMI		90
** **<25	46 (51.1%)	
** **≥25	44 (48.9%)	

Smoking		90
** **Yes	34 (37.8%)	
** **No	56 (62.2%)	

COBB		90
** **<10°	58 (64.4%)	
** **≥10°	32 (35.6%)	

cSVA	7.8 (6.25–9.15)	89
** ** *T*1 slope	34.2 (30.05–38)	

Fracture site	6 (2–8)	90
** **Single	27 (30%)	
** **Multi	63 (70%)	

Fracture type		90
** **I	28 (31.1%)	
** **II	30 (33.3%)	
** **III	32 (35.6%)	

Subtype		90
** **A	37 (41.1%)	
** **B	36 (40%)	
** **C	17 (18.9%)	

Preoperative ASIA		90
** **A, B, C	47 (52.2%)	
** **D, E	43 (47.8%)	

Preoperative mJOA		90
** **Operation time	155 (120–210)	81
** **Blood loss	300 (150–500)	81

Treatment		90
** **PF	48 (53.3%)	
** **PF + AF	3 (3.3%)	
** **Conservative treatment	8 (8.9%)	
** **AF	31 (34.4%)	

**Table 2:** Logistic and ASIA.

Variable	Unadjusted OR (95% CI)	*P* value	Adjusted OR (95% CI)	*P* value
Sex (female vs. male)	0.51 (0.1–2.63)	0.423		
Age (≥60 vs. <60)	7.28 (2.42–21.88)	0	2.31 (0.35–15.21)	0.383
BMI (≥25 vs. <25)	0.3 (0.12–0.76)	0.011	0.26 (0.06–1.2)	0.084
Smoking (yes vs. no)	0.69 (0.28–1.73)	0.435		
COBB score (≥10° vs. <10°)	1.01 (0.41–2.49)	0.992		
cSVA (every 1 increment)	1.2 (1–1.43)	0.051	1.23 (0.89–1.69)	0.205
*T*1 slope (every 1 increment)	0.94 (0.87–1.03)	0.187		
Fracture site (single vs. multiple)	0.16 (0.04–0.57)	0.005	—	—
Fracture type		<0.001		0.821
** **I	Reference		Reference	
** **II	0.33 (0.08–1.45)	0.142	—	—
** **III	5.73 (1.86–17.63)	0.002	1.8 (0.29–11.35)	0.53
Subtype		0.072		0.869
** **A	Reference		Reference	
** **B	1.19 (0.43–3.28)	0.739	1 (0.17–6.08)	0.997
** **C	3.86 (1.15–12.91)	0.029	1.64 (0.23–11.44)	0.62
ASIA (A, B, and C vs. D and E)	74.12 (9.35–587.66)	<0.001	40.52 (3.79–433.73)	0.002
mJOA (every 1 increment)	0.68 (0.57–0.81)	<0.001	0.91 (0.69–1.2)	0.495
Treatment				
** **AF	Reference	0.741		
** **PF	1.34 (0.51–3.56)	0.556		
** **PF + AF	1.22 (0.1–15.23)	0.876		
** **Conservative treatment	2.44 (0.5–11.97)	0.27		

**Table 3:** Logistic and mJOA.

Variable	Unadjusted OR (95% CI)	*P* value	Adjusted OR (95% CI)	*P* value
Sex (female vs. male)	1 (0.25–4)	1		
Age (≥60 vs. <60)	10.69 (2.3–49.59)	0.002	4.73 (0.45–49.46)	0.194
BMI (≥25 vs. <25)	0.76 (0.33–1.74)	0.51		
Smoking (yes vs. no)	0.47 (0.2–1.13)	0.091	0.24 (0.06–0.95)	0.043
COBB score (≥10° vs. <10°)	0.96 (0.4–2.28)	0.922		
cSVA (every 1 increment)	0.95 (0.8–1.11)	0.507		
*T*1 slope (every 1 increment)	0.99 (0.92–1.07)	0.821		
Fracture site (single vs. multiple)	0.21 (0.08–0.56)	0.002	0.05 (0–1.28)	0.07
Fracture type		0.003		0.538
** **I	Reference		Reference	
** **II	0.33 (0.08–1.45)	0.142	3.68 (0.15–88.42)	0.421
** **III	5.73 (1.86–17.63)	0.002	0.61 (0.1–3.56)	0.578
Subtype		0.001		0.053
** **A	Reference	0.04	Reference	
** **B	1.19 (0.43–3.28)	0.739	2.29 (0.56–9.37)	0.25
** **C	3.86 (1.15–12.91)	0.029	16.73 (1.62–172.93)	0.018
ASIA (A, B, and C vs. D and E)	8.54 (3.29–22.18)	<0.001	2.93 (0.75–11.45)	0.123
mJOA (every 1 increment)	0.64 (0.54–0.77)	<0.001	0.65 (0.5–0.84)	0.001
Treatment				
** **AF	Reference	0.866		
** **PF	1.06 (0.43–2.63)	0.902		
** **PF + AF	0.41 (0.03–5.03)	0.487		
** **Conservative treatment	1.37 (0.28–6.78)	0.697		

**Table 4:** COX and OS.

Variable	Unadjusted HR (95% CI)	*P* value	Adjusted HR (95% CI)	*P* value
Sex (female vs. male)	0.04 (0–130.87)	0.44		
Age (≥60 vs. <60)	13.09 (3.53–48.49)	0	2.93 (0.6–14.32)	0.185
BMI (≥25 vs. <25)	0.46 (0.14–1.53)	0.205		
Smoking (yes vs. no)	1.2 (0.38–3.78)	0.756		
COBB score (≥10° vs. <10°)	0.38 (0.12–1.21)	0.101		
cSVA (every 1 increment)	1.01 (0.81–1.26)	0.931		
*T*1 slope (every 1 increment)	0.95 (0.85–1.07)	0.381		
Fracture site (single vs. multiple)	0.2 (0.03–1.55)	0.124		
Fracture type		0.01		0.59
** **I	Reference		Reference	
** **II	0.95 (0.06–15.17)	0.971	0.55 (0.03–11.43)	0.701
** **III	10.33 (1.32–80.74)	0.026	1.65 (0.14–19.59)	0.69
Subtype		0.001		0.085
** **A	Reference		Reference	
** **B	3.16 (0.33–30.33)	0.32	3.01 (0.3–29.73)	0.346
** **C	21.47 (2.68–171.94)	0.004	9.24 (1.04–82.06)	0.046
ASIA (A, B, and C vs. D and E)	11.46 (1.48–88.85)	0.02	1.46 (0.11–18.56)	0.773
mJOA (every 1 increment)	0.63 (0.47–0.83)	0.001	0.77 (0.53–1.11)	0.155
Treatment		0.741		
** **AF	Reference			
** **PF	172324.78 (0–2.994*E* + 144)	0.941		
** **PF + AF	—	—		
** **Conservative treatment	386706.5 (0–6.727*E* + 144)	0.937		

## Data Availability

The basic information of clinical objects data used to support the findings of this study is included within the supplementary information file. Detailed imaging information and original case images can be obtained from the corresponding authors and provided by e-mail after approval by the local ethics committee.

## Abbreviations

AS: Ankylosing spondylitis
mJOA: Modified Japanese Orthopaedic Association
ASIA: American Spinal Cord Injury Association
AF: Anterior cervical decompression and fusion
PF: Posterior cervical decompression and fusion.

## Supplementary Materials

Supplementary Materials In Section 3.4, the article shows the statistical table of 90 patients selected for prospective analysis, including 81 males and 9 females. We summarize and count the specific information of 90 patients including “sex, age, BMI, SMOK, C_2-7_ COBB, cSVA, *T*1 slope, fracture site, fracture type, ASIA, mJOA, treatment, fixation level, operation time, blood loss, fusion, last follow-up time, ASIA at last follow-up, mJOA at last follow-up, and complication,” a total of 20 indicators, see Table S1 for details.
