# Supplementary material for: Classification and Treatment for Cervical Spine Fracture with Ankylosing Spondylitis: A Clinical Nomogram Prediction Study
Source: Pain Res Manag. 2022 Mar 4;2022:7769775. doi: 10.1155/2022/7769775 (PMC8916892; doi:10.1155/2022/7769775)
Supplement: Supplementary Materials — In Section 3.4, the article shows the statistical table of 90 patients selected for prospective analysis, including 81 males and 9 females. We summarize and count the specific information of 90 patients including “sex, age, BMI, SMOK, C2-7 COBB, cSVA, T1 slope, fracture site, fracture type, ASIA, mJOA, treatment, fixation level, operation time, blood loss, fusion, last follow-up time, ASIA at last follow-up, mJOA at last follow-up, and complication,” a total of 20 indicators, see Table S1 for details. [file 7769775.f1.docx]

In the section "3.4 Basic Information of Prospective Studies", the article shows the statistical table of 90 patients selected for prospective analysis, including 81 males and 9 females.The basic information of clinical objects data used to support the findings of this study are included in this supplementary information file(s).

We summarize and count the specific information of 90 patients including: "Sex, Age, BMI, SMOK, C2-7 COBB, cSVA, T1 slope, Fracture site, Fracture type, ASIA,mJOA ,Treatment, Fixation level, Operation time, Blood loss, Fusion, Last follow- up time, ASIA at Last Follow-up, mJOA at Last Follow-up, Complication”, a total of 20 indicators,see the table below for details.

| Case No. | Sex | Age | BMI | SMOK | C2-7COBB（°） | cSVA（mm） | T1 slope（°） | Fracture site | Fracture type | ASIA | mJOA | Treatment | Fixation level | Operation time(min) | Blood loss(ml) | Fusion | Last follow-up time（month） | ASIA at Last Follow-up | mJOA at Last Follow-up | Complication |
| --- | --- | --- | --- | --- | --- | --- | --- | --- | --- | --- | --- | --- | --- | --- | --- | --- | --- | --- | --- | --- |
| 1 | Male, | 61 | 16.7 | Y | -11.2 | 7.2 | 27.1 | C6-C7 | IA | C | 2 | AF | C5-C7 | 145 | 200 | yes | 36 | D | 11 |  |
| 2 | Male, | 38 | 22.3 | N | -10.7 | 6.3 | 27.3 | C7 | IIA | D | 5 | PF | C5-T1 | 185 | 500 | yes | 29 | E | 16 |  |
| 3 | Male, | 56 | 21.7 | Y | -3.1 | 6.5 | 28.4 | C6-C7 | IIIA | D | 5 | AF | C5-T1 | 90 | 100 | yes | 16 | D | 14 | screw loosing |
| 4 | Male, | 44 | 27.6 | Y | -12.2 | 7.3 | 29.1 | C7 | IIA | D | 9 | PF | C6-T1 | 230 | 500 | yes | 31 | E | 15 |  |
| 5 | Male, | 77 | 19.4 | N | -7.3 | 8.4 | 33.2 | C6-C7 | IIIC | B | 2 | PF | C5-T2 | 210 | 400 | N/A | 5 | B | 1 | Died pneumonia |
| 6 | Male, | 57 | 22.8 | Y | -22.6 | 8.5 | 33.1 | C5-C6 | IA | D | 4 | AF | C4-C7 | 305 | 800 | yes | 29 | D | 14 |  |
| 7 | Male, | 47 | 24.2 | Y | -13.1 | 9.1 | 31.1 | C6-C7 | IB | B | 0 | AF | C5-T1 | 105 | 200 | yes | 7 | B | 4 | hematoma |
| 8 | Male, | 29 | 17.9 | N | -9.2 | 10.1 | 28.5 | C5-C6 | IIIB | B | 2 | PF | C3-C7 | 270 | 500 | yes | 33 | C | 11 |  |
| 9 | Male, | 70 | 18.6 | Y | -11.1 | 6.3 | 28.4 | C4 | IIA | B | 2 | Conservative treatment |  |  |  | N/A | 24 | D | 12 |  |
| 10 | Male, | 47 | 23.1 | N | -8.2 | 6.5 | 27.6 | C6-C7 | IIIB | C | 8 | PF+AF | PF:C3-T2 | 270 | 800 | yes | 21 | D | 16 |  |
|  |  |  |  |  |  |  |  |  |  |  |  |  | AF:C6-C7 |  |  |  |  |  |  |  |
| 11 | Male, | 61 | 21.6 | Y | -9.6 | 5.7 | 30.1 | C6 | IIA | D | 9 | AF | C4-T1 | 110 | 120 | yes | 34 | E | 16 |  |
| 12 | Male, | 53 | 25.5 | Y | -10.4 | 8.3 | 27.4 | C4-C5 | IB | D | 5 | AF | C3-C6 | 130 | 50 | yes | 34 | D | 14 |  |
| 13 | Male, | 45 | 26.2 | N | -10.9 | 9.2 | 29.1 | C6-C7 | IIIA | D | 8 | PF | C4-T2 | 280 | 1500 | yes | 34 | D | 16 |  |
| 14 | Male, | 47 | 25.1 | N | -11.7 | 9.7 | 29.8 | C4-C5 | IB | D | 7 | PF | C2-C7 | 260 | 1200 | yes | 34 | D | 14 |  |
| 15 | Male, | 46 | 19.8 | Y | -8.4 | 6.3 | 22.7 | C6-C7 | IIIC | B | 1 | Conservative treatment |  |  |  | N/A | 3.5 | A | 0 | Died,respiratory failure |
| 16 | Male, | 65 | 22.3 | N | -12.3 | 10.1 | 31.7 | C6-C7 | IIIB | C | 1 | PF | C4-T2 | 110 | 400 | yes | 17 | A | 0 | skin ulceration |
| 17 | Female, | 49 | 21.5 | N | -9.8 | 5.4 | 35.3 | C6 | IIA | D | 9 | PF | C4-C7 | 120 | 150 | yes | 22 | D | 15 |  |
| 18 | Male, | 45 | 25.8 | Y | -21.2 | 4.1 | 35.6 | C5 | IIC | D | 8 | PF | C3-C7 | 185 | 100 | yes | 28 | D | 14 | CSF leakage |
| 19 | Male, | 51 | 26.2 | Y | -13.2 | 3.8 | 36.7 | C6-C7 | IA | D | 9 | AF | C5-T1 | 115 | 100 | yes | 28 | D | 14 |  |
| 20 | Male, | 50 | 20.3 | N | -11.7 | 6.7 | 38.3 | C5-C6 | IB | D | 9 | AF | C4-C7 | 110 | 100 | yes | 36 | E | 15 |  |
| 21 | Male, | 52 | 22.7 | N | -7.3 | 7.9 | 35.2 | C5-C6 | IA | D | 10 | AF | C5-C7 | 80 | 50 | yes | 36 | E | 15 |  |
| 22 | Male, | 45 | 21 | Y | -8.5 | 9.2 | 34.2 | C7 | IIB | D | 11 | PF | C4-T2 | 230 | 300 | yes | 36 | E | 15 |  |
| 23 | Male, | 51 | 24.5 | N | -6.4 | 8.4 | 31 | C7 | IIA | D | 12 | PF | C5-T2 | 160 | 100 | yes | 36 | E | 16 |  |
| 24 | Male, | 38 | 19.5 | N | -13.1 | 11.0 | 27.9 | C5-C6 | IB | D | 8 | AF | AF:C5-C6 | 110 | 120 | yes | 30 | E | 12 | screw pullout |
|  |  |  |  |  |  |  |  |  |  |  |  | Revision:AF+PF | Revision | 275 | 200 |  |  |  |  |  |
|  |  |  |  |  |  |  |  |  |  |  |  |  | AF: C4-C6 |  |  |  |  |  |  |  |
|  |  |  |  |  |  |  |  |  |  |  |  |  | PF:C4-T1 |  |  |  |  |  |  |  |
| 25 | Male, | 38 | 21.6 | N | -5.8 | 6.2 | 38.9 | C5-C6 | IIIC | C | 6 | AF | C3-C7 | 100 | 110 | yes | 22 | E | 9 |  |
| 26 | Female, | 65 | 24.8 | N | -12.4 | 7.3 | 39.6 | C7 | IIA | B | 1 | PF | C4-T2 | 170 | 500 | yes | 7 | D | 6 | hematoma |
| 27 | Male, | 54 | 30.9 | Y | -13.7 | 8.9 | 40.1 | C6 | IIB | D | 11 | PF | C5-T2 | 235 | 300 | yes | 24 | E | 14 |  |
| 28 | Male, | 57 | 32.5 | N | -14.3 | 17 | 30.2 | C5-C6 | IIIA | A | 1 | PF | C3-C7 | 230 | 150 | yes | 3 | C | 8 | hematoma |
| 29 | Male, | 40 | 26.3 | N | -16.2 | 13 | 28.4 | C5-C6 | IA | D | 5 | AF | C4-C7 | 90 | 50 | yes | 36 | D | 12 |  |
| 30 | Male, | 51 | 26.4 | Y | -7.9 | 8.2 | 37.9 | C6-C7 | IA | D | 2 | AF | C5-T1 | 160 | 200 | yes | 36 | D | 10 |  |
| 31 | Male, | 55 | 31.1 | N | -6.7 | 8.1 | 43.1 | C6-C7 | IIIA | B | 5 | AF | C5-T1 | 90 | 100 | yes | 14 | B | 14 | DVT |
| 32 | Male, | 39 | 21.4 | N | -22.7 | 7.4 | 43.8 | C5-C6 | IB | D | 8 | Conservative treatment |  |  |  | N/A | 4 | C | 8 | Died pneumonia |
| 33 | Male, | 76 | 22.6 | N | -8.7 | 6.3 | 37.2 | C6-C7 | IIIC | B | 2 | PF | C5-T2 | 170 | 400 | N/A | 9.5 | B | 1 | Died, skin ulceration and fracture nonunion |
| 34 | Male, | 58 | 21.7 | Y | -17.8 | 13.2 | 38.3 | C5-C6 | IA | C | 8 | AF | C4-C7 | 150 | 200 | yes | 32 | C | 13 |  |
| 35 | Male, | 45 | 18.3 | N | -18.3 | 11 | 34.2 | C7 | IIA | D | 6 | PF | C6-T1 | 210 | 500 | yes | 36 | D | 15 |  |
| 36 | Male, | 49 | 18.8 | N | -10.3 | 10..5 | 23.7 | C6-C7 | IB | B | 1 | AF | C5-C7 | 140 | 260 | yes | 36 | B | 4 |  |
| 37 | Male, | 31 | 22.5 | Y | -12.1 | 14.2 | 31.9 | C5-C6 | IIIB | B | 3 | PF | C3-C7 | 270 | 500 | yes | 18 | C | 7 | CSF leakage |
| 38 | Male, | 43 | 25.6 | N | -13.7 | 9.2 | 33.7 | C6-C7 | IIIB | C | 8 | Conservative treatment |  |  |  | yes | 36 | D | 11 |  |
| 39 | Male, | 60 | 26.3 | Y | -17.9 | 8.7 | 34.8 | C6 | IIA | D | 10 | PF | C4-T1 | 140 | 220 | yes | 36 | E | 16 |  |
| 40 | Male, | 44 | 27.2 | Y | -14.6 | 8.5 | 35.1 | C4-C5 | IB | C | 7 | AF | C3-C6 | 130 | 250 | yes | 36 | D | 10 |  |
| 41 | Male, | 46 | 19.4 | N | -7.5 | 6.2 | 40.2 | C6-C7 | IIIA | D | 8 | PF | C4-T2 | 280 | 1500 | yes | 24 | D | 16 |  |
| 42 | Male, | 64 | 19.9 | N | -8.3 | 5.2 | 41.7 | C6-C7 | IIIB | C | 2 | PF | C4-T2 | 210 | 600 | N/A | 1.7 | B | 0 | Died, CSF leakage and SAH |
| 43 | Male, | 50 | 21.3 | N | -3.2 | 5.8 | 33.8 | C6-C7 | IIA | C | 9 | PF | C4-T1 | 180 | 750 | yes | 28 | D | 12 |  |
| 44 | Male, | 46 | 22.6 | Y | -12.3 | 3.3 | 37.9 | C5 | IIC | D | 6 | PF | C3-C7 | 165 | 800 | yes | 7 | D | 14 | hematoma |
| 45 | Male, | 50 | 30.5 | Y | -12.4 | 2.7 | 44.8 | C6-C7 | IA | D | 9 | AF | C5-T1 | 115 | 200 | yes | 36 | D | 10 |  |
| 46 | Male, | 52 | 29.4 | N | -11.7 | 2.9 | 42.3 | C5-C6 | IB | D | 6 | AF | C4-C7 | 140 | 250 | yes | 36 | D | 11 |  |
| 47 | Male, | 46 | 28.3 | N | -8.8 | 3.5 | 47.2 | C7 | IIB | C | 11 | PF | C4-T2 | 230 | 600 | yes | 36 | E | 12 |  |
| 48 | Male, | 50 | 25.2 | N | -10.6 | 4.8 | 45.6 | C7 | IIA | D | 8 | PF | C5-T2 | 190 | 400 | yes | 36 | E | 10 |  |
| 49 | Male, | 39 | 26.1 | N | -13.2 | 7.2 | 37.8 | C5-C6 | IB | C | 4 | AF | C4-C7 | 140 | 120 | yes | 36 | D | 9 |  |
| 50 | Male, | 67 | 25.8 | Y | -16.3 | 8.3 | 33.2 | C5-C6 | IIIC | C | 5 | Refuse surgery |  |  |  | N/A | 0.5 | B | 3 | Died, progressive neurological injury |
| 51 | Male, | 55 | 25.5 | N | -7.3 | 8.9 | 29.9 | C6 | IIB | C | 8 | PF | C5-T2 | 190 | 700 | yes | 36 | C | 8 |  |
| 52 | Male, | 63 | 26.1 | Y | -7.6 | 7.6 | 30.6 | C5-C6 | IIIA | B | 1 | PF | C3-C7 | 230 | 150 | N/A | 2 | B | 0 | Died, respiratory failure |
| 53 | Male, | 42 | 22.9 | N | -11.6 | 9.1 | 40.1 | C5-C6 | IA | C | 5 | AF | C4-C7 | 130 | 250 | yes | 36 | C | 6 |  |
| 54 | Male, | 58 | 23.6 | Y | -15.3 | 10.2 | 26.8 | C5-C6 | IA | C | 2 | AF | C5-C6 | 115 | 100 | yes | 36 | C | 6 |  |
| 55 | Male, | 59 | 24.4 | N | -15.8 | 7.7 | 27.4 | C6-C7 | IIIA | B | 2 | PF | C4-T2 | 120 | 250 | yes | 36 | C | 6 |  |
| 56 | Male, | 43 | 24.6 | N | -12.9 | 8.4 | 23.1 | C4-C5 | IIIB | A | 0 | PF | C4-T2 | 135 | 280 | N/A | 1 | A | 0 | Died, respiratory failure |
| 57 | Male, | 76 | 23.1 | N | -14.2 | 9.6 | 28.6 | C6-C7 | IIIC | B | 2 | PF | C4-T2 | 125 | 300 | N/A | 1.5 | A | 0 | Died, respiratory failure |
| 58 | Male, | 60 | 25.2 | Y | -9.9 | 5.8 | 40.3 | C4 | IIA | C | 3 | Conservative treatment |  |  |  | yes | 24 | D | 12 |  |
| 59 | Male, | 50 | 25.7 | Y | -11.5 | 7.4 | 40.1 | C5-C6 | IIIA | D | 8 | PF | C3-T1 | 145 | 450 | yes | 24 | D | 16 |  |
| 60 | Male, | 68 | 26.1 | Y | -22.1 | 8.3 | 37.4 | C6-C7 | IIIC | B | 2 | PF | C5-T2 | 135 | 300 | N/A | 6 | B | 0 | Died pneumonia |
| 61 | Fenmal, | 62 | 30.1 | N | -20.3 | 7.7 | 37.8 | C6-C7 | IIIB | C | 2 | PF | C4-T2 | 125 | 350 | yes | 32 | C | 6 |  |
| 62 | Male, | 56 | 26.7 | N | -17.6 | 7.6 | 36.9 | C6 | IIB | D | 10 | PF | C5-T2 | 235 | 250 | yes | 34 | E | 12 |  |
| 63 | Fenmal, | 46 | 27.3 | Y | -15.4 | 11.3 | 40.4 | C5 | IIC | D | 8 | PF | C3-C7 | 150 | 300 | yes | 34 | D | 14 |  |
| 64 | Male, | 42 | 26.5 | Y | -16.3 | 14.8 | 32.7 | C6-C7 | IB | D | 10 | AF | AF:C6-C7 | 110 | 120 | yes | 34 | E | 12 |  |
| 65 | Male, | 40 | 26.4 | N | -16.9 | 13.9 | 33.6 | C5-C6 | IIIB | C | 10 | PF+AF | PF:C3-T1 | 220 | 650 | yes | 36 | D | 14 |  |
|  |  |  |  |  |  |  |  |  |  |  |  |  | AF:C5-C6 |  |  |  |  |  |  |  |
| 66 | Male, | 75 | 27.3 | N | -7.9 | 11.3 | 29.6 | C6-C7 | IIIC | A | 0 | PF | C5-T2 | 210 | 400 | N/A | 3 | A | 0 | Died pneumonia |
| 67 | Male, | 39 | 25.8 | Y | -4.2 | 10.4 | 33.3 | C5-C6 | IB | C | 3 | AF | C5-C6 | 170 | 180 | yes | 36 | D | 12 |  |
| 68 | Male, | 50 | 25.7 | N | -6.3 | 8.4 | 34.7 | C6 | IIB | C | 10 | PF | C4-T1 | 210 | 400 | yes | 36 | D | 12 |  |
| 69 | Male, | 45 | 26.3 | N | -17.2 | 8.9 | 35.1 | C7 | IIA | D | 6 | PF | C6-T1 | 180 | 320 | yes | 36 | E | 12 |  |
| 70 | Male, | 62 | 27.1 | N | -12.6 | 7.4 | 39.4 | C6-C7 | IIIA | B | 1 | AF | C5-T1 | 145 | 250 | yes | 32 | C | 8 |  |
| 71 | Female, | 52 | 27.4 | Y | -11.9 | 7.2 | 37.7 | C5 | IIB | D | 3 | PF | C3-C7 | 120 | 600 | yes | 34 | E | 13 |  |
| 72 | Male, | 46 | 27.8 | N | -8.3 | 5.7 | 36.4 | C5-C6 | IB | D | 5 | AF | C4-C7 | 120 | 420 | yes | 36 | D | 8 |  |
| 73 | Male, | 57 | 19.5 | N | -9.3 | 5.9 | 38.3 | C6-C7 | IA | D | 4 | AF | C6-C7 | 130 | 150 | yes | 24 | D | 9 |  |
| 74 | Male, | 49 | 19.9 | N | -12.1 | 6.8 | 35.1 | C5-C6 | IIIA | B | 8 | AF | C5-C6 | 110 | 130 | yes | 24 | C | 10 |  |
| 75 | Male, | 64 | 21.4 | N | -14.5 | 6.3 | 37.2 | C5-C6 | IIIA | C | 7 | AF | C4-C7 | 130 | 110 | yes | 28 | C | 9 |  |
| 76 | Male, | 58 | 26.2 | N | -17.6 | 7.8 | 33.4 | C5 | IIB | D | 6 | PF | C3-C7 | 140 | 530 | yes | 32 | D | 8 |  |
| 77 | Male, | 66 | 24.6 | Y | -17.2 | 9.4 | 30.8 | C5-C7 | IIIC | D | 9 | PF | C5-T1 | 190 | 990 | yes | 36 | D | 10 |  |
| 78 | Female, | 31 | 23.6 | N | -22.1 | 8.4 | 31.2 | C5-C6 | IB | E | 10 | Conservative treatment |  |  |  | N/A | 24 | E | 13 |  |
| 79 | Male, | 66 | 23.3 | N | -8.6 | 8.9 | 29.7 | C6 | IIC | C | 3 | PF | C3-C7 | 170 | 260 | yes | 24 | C | 7 |  |
| 80 | Male, | 52 | 19.4 | N | -9.4 | 7.2 | 29.8 | C5 | IIC | D | 6 | PF | C3-C7 | 160 | 370 | yes | 20 | D | 9 |  |
| 81 | Male, | 46 | 33 | N | -7.7 | 5.3 | 30.5 | C6-C7 | IIIC | B | 3 | PF | C3-C7 | 180 | 410 | yes | 15 | D | 9 |  |
| 82 | Male, | 39 | 29.9 | Y | -12.6 | 5.1 | 31.1 | C5-C6 | IB | B | 9 | Conservative treatment |  |  |  | N/A | 18 | C | 11 |  |
| 83 | Female, | 48 | 25.6 | N | -11.7 | 5.4 | 32.6 | C4-C5 | IIA | C | 5 | PF | C2-C7 | 210 | 1100 | Yes | 22 | D | 7 |  |
| 84 | Male, | 56 | 25.9 | Y | -14.9 | 4.2 | 35.6 | C5-C6 | IIA | D | 7 | PF | C3-C7 | 190 | 520 | Yes | 32 | D | 9 |  |
| 85 | Male, | 47 | 26.2 | N | -20.5 | 8.5 | 40.8 | C5-C6 | IB | D | 8 | AF | C4-C7 | 210 | 105 | Yes | 36 | D | 12 |  |
| 86 | Male, | 54 | 25.4 | N | -20.1 | 6.2 | 41.9 | C5-C6 | IB | C | 5 | AF | C4-C7 | 105 | 110 | Yes | 32 | D | 9 |  |
| 87 | Female, | 48 | 24.1 | N | -22.3 | 6 | 41.5 | C6-C7 | IIIC | B | 9 | PF+AF |  |  |  | Yes | 34 | C | 11 |  |
| 88 | Male, | 70 | 18.3 | Y | -6.9 | 8.1 | 37.5 | C5 | IIC | A | 3 | PF | C3-C7 | 107 | 430 | Yes | 1.5 | A | 0 | Died pneumonia |
| 89 | Female, | 51 | 25.3 | N | -4.8 | 9.2 | 34.8 | C6 | IIB | D | 1 | PF | C4-T1 | 190 | 750 | Yes | 32 | D | 6 |  |
| 90 | Male, | 61 | 28.7 | N | -7.3 | 10 | 32.9 | C4-C5 | IIIB | D | 4 | PF | C3-C7 | 155 | 450 | Yes | 32 | D | 7 |  |
